# Measurement of Ex Vivo Liver, Brain and Pancreas Thermal Properties as Function of Temperature

**DOI:** 10.3390/s21124236

**Published:** 2021-06-21

**Authors:** Ahad Mohammadi, Leonardo Bianchi, Somayeh Asadi, Paola Saccomandi

**Affiliations:** Department of Mechanical Engineering, Politecnico di Milano, 20156 Milan, Italy; ahad.mohammadi@polimi.it (A.M.); leonardo.bianchi@polimi.it (L.B.); somayeh.asadi@polimi.it (S.A.)

**Keywords:** thermal properties, temperature dependence, ex vivo study, pancreas, brain, liver

## Abstract

The ability to predict heat transfer during hyperthermal and ablative techniques for cancer treatment relies on understanding the thermal properties of biological tissue. In this work, the thermal properties of ex vivo liver, pancreas and brain tissues are reported as a function of temperature. The thermal diffusivity, thermal conductivity and volumetric heat capacity of these tissues were measured in the temperature range from 22 to around 97 °C. Concerning the pancreas, a phase change occurred around 45 °C; therefore, its thermal properties were investigated only until this temperature. Results indicate that the thermal properties of the liver and brain have a non-linear relationship with temperature in the investigated range. In these tissues, the thermal properties were almost constant until 60 to 70 °C and then gradually changed until 92 °C. In particular, the thermal conductivity increased by 100% for the brain and 60% for the liver up to 92 °C, while thermal diffusivity increased by 90% and 40%, respectively. However, the heat capacity did not significantly change in this temperature range. The thermal conductivity and thermal diffusivity were dramatically increased from 92 to 97 °C, which seems to be due to water vaporization and state transition in the tissues. Moreover, the measurement uncertainty, determined at each temperature, increased after 92 °C. In the temperature range of 22 to 45 °C, the thermal properties of pancreatic tissue did not change significantly, in accordance with the results for the brain and liver. For the three tissues, the best fit curves are provided with regression analysis based on measured data to predict the tissue thermal behavior. These curves describe the temperature dependency of tissue thermal properties in a temperature range relevant for hyperthermia and ablation treatments and may help in constructing more accurate models of bioheat transfer for optimization and pre-planning of thermal procedures.

## 1. Introduction

Over the last 30 years, hyperthermal and ablative procedures have been studied as an alternative to surgery in cancer treatment. Different techniques based on thermal processes, e.g., laser ablation, microwave ablation, radiofrequency ablation and focused ultrasound, have been investigated for the hyperthermal treatment of cancer [1]. These methods rely on the localized increase in the tissue temperature above physiological temperature thresholds to induce thermal damage in the cells and coagulative necrosis. Augmented temperatures produce effects on cells in several modalities. Indeed, the degree of thermal damage can be classified according to the local tissue temperature and duration of treatment at a given temperature [2]. Hyperthermia starts in the temperature range between 42 and 45 °C; at 50 °C, the reduction in enzymatic activity begins; at 60 °C, the denaturation of proteins, coagulation of collagen and membrane permeabilization rapidly occur, leading to a cytotoxic effect and coagulative necrosis, which is the primary cause of cell death during thermal ablation of tumors; and for temperatures close to 100 °C and above, the effects of vaporization and tissue carbonization befall [3,4]. Hence, a temperature range of 42 to 100 °C is of interest for implementing the different techniques available for cancer treatment, from hyperthermia to thermal ablation [5].

Despite promising results, the principal limitation to the widespread adoption of thermal techniques in clinical settings is still related to the difficulty to guarantee a complete tumor ablation while sparing healthy tissue. From the technological point of view, mathematical modeling for treatment pre-planning has been developed to simulate the tissue temperature profile and therefore increase the treatment efficacy and safety. In this context, information of the tissue thermal properties as a function of temperature is necessary for the accurate prediction of the thermal outcome. Indeed, the result of hyperthermic therapies is strictly related to the temperature distribution of the treated biological tissues. This is, in turn, influenced by the delivery modality of the thermal dose and the intrinsic physical properties of the tissue, such as the tissue thermal properties, which vary according to temperature due to thermal-induced structural modifications occurring during treatments [6]. Various computational models that require thermal properties to solve the governing equations have been developed in recent years [7,8,9,10,11,12,13]. The most common approach for describing the heat transfer in tissues relies on Pennes’ equation (Equation (1)):

(1) $$\rho C\frac{\partial T}{\partial t}=\nabla \left(k\nabla T\right)+Q_{s}+Q_{b}+Q_{met}$$

where *ρ* (kg/m^3^) is the tissue density, *C* (J/kg·K) is the heat capacity, *k* (W/(m·K)) is the thermal conductivity, *T* (K) is the transient temperature, *Q_s_* (W/m^3^) is the external source used to induce the thermal treatment and *Q_b_* (W/m^3^) and *Q_met_* (W/m^3^) are the terms related to heat dissipation caused by the blood flow and the metabolic heat, respectively. The thermophysical behavior of tissues in the heat transfer phenomenon can be synthesized in a single parameter, i.e., the thermal diffusivity D (m^2^/s), which is defined as D $=\frac{k}{\rho C}$. The thermal properties characterize the ability of materials to conduct, transfer, store and release heat [14]. The accuracy of the model in Equation (1) is highly dependent on the accurate definition of the thermophysical properties of the target tissue, as it has already been proved by several studies [15,16]. Therefore, replacing the thermal parameters that are generally considered constant values—usually at room temperature—with temperature-dependent physical parameters can lead to a more accurate prediction of the treatment outcome [17].

Many scientific studies have been presented to measure the thermal properties of biological tissues. These studies are mainly focused on liver tissue [18,19,20,21] and muscles [22,23,24,25], and some data are reported for other organs, such as the kidney [26] and brain [27]. However, as mentioned before, most of these studies measured the thermal properties at a constant or low temperature which is not completely appropriate for thermal ablation modeling. One of the first and more extensive studies on the temperature dependency of tissue thermal properties was performed by Valvano et al. in 1985. The authors used a self-heated thermistor to determine the thermal conductivity and diffusivity of ex vivo kidneys, spleens, livers, brains, hearts, lungs, pancreases, colon cancers and breast cancers within a temperature range between 3 and 45 °C [28]. Within this range, the measured properties were slightly temperature-dependent and showed a weak linear increase with temperature. The authors observed a significant inter-tissue variation in thermal diffusivity and conductivity, as well as a match between tissue thermal diffusivity and water thermal diffusivity.

More recent studies have started to measure the tissue-specific heat capacity, thermal diffusivity and conductivity up to the ablative temperatures. Among all the organs, the liver is the most investigated due to the high demand for ablative therapies for liver disease treatment [29]. Choi et al. measured the specific heat capacity and the thermal conductivity for ex vivo human and porcine livers from 20 to 85 °C. The thermal conductivity and specific heat capacity increased by 12% at 70 °C according to their research [30]. Lopresto et al. observed that the thermal properties did not significantly vary with temperature until 90 °C; after this temperature, thermal properties exponentially increased until the water phase transition process [19]. Nuno P. Silva et al. investigated the thermal properties of ex vivo ovine livers at temperatures ranging from 25 to 97 °C. This study reported a significant increase in thermal properties only above 90 °C [20]. The authors also measured the thermal properties of different biological tissues, considering the influence of their density and water content [31]. Haemmerich et al. measured the specific heat of liver tissue in vitro in the range of 25.0–83.5 °C. They found that the liver specific heat increased by 17% at 83.5 °C, compared to temperatures below 65 °C [32]. Guntur et al. measured thermal properties in ex vivo porcine livers heated up to 90 °C and then cooled to 20 °C. The thermal conductivity decreased by 9.6% from its initial value (20 °C) at the turning temperature (35 °C) and rose by 45% at 90 °C from its minimum (35 °C) [18]. Except for the liver, there is little information in the literature about other tissues that are clinical targets of ablative procedures, such as the brain [33] and the pancreas [34].

Regarding the brain, Salcman et al. measured the heat capacity of the brain as a function of the cerebral blood flow and temperature from 20 to 49 °C for adult dogs [27]. Cooper and Trezek reported the thermal properties of the brain white and gray parts at temperatures ranging from 5 to 20 °C [35], and Bowman measured thermal properties of the brain at body temperature, i.e., 37 °C [36]. 

Considering the pancreas, the study of Valvano et al. is one of the few reports about the thermal properties of this organ [28], along with a recent report on the specific heat capacity measurement in the temperature range from −160 to 40 °C, using differential scanning calorimetry [37].

Considering the limited information for the brain and pancreas and increasing interest of the biomedical community in thermal therapy on these two organs, this study aims to measure the thermal properties of the brain and pancreas as a function of temperature.

We measured the thermal diffusivity, thermal conductivity and volumetric specific heat of healthy and ex vivo calf brains and porcine pancreases with a commercial analyzer with a dual-needle sensor, which has been proved to be suitable to measure the thermal properties of tissue samples. The experimental approach used in our work was firstly validated on ex vivo porcine livers, and the results are compared to the data provided by previous studies. As a result, the best fit curves are presented based on measured data, with the aim to propose the tissue-specific model of the thermal properties as a function of the therapeutic temperature.

## 2. Materials and Methods

### 2.1. Tissue Preparation and Experimental Setup

Thermal properties were measured with a commercial analyzer (TEMPOS, Meter Group, Inc., Pullman, WA, USA, accuracy: 10%) with an SH-3 dual-needle sensor, which has already been approved for this aim [19,20,31,38]. The needles of this measurement system are 30 mm long, 6 mm spaced, 1.3 mm in diameter and could measure thermal conductivity, volumetric heat capacity and thermal diffusivity in non-liquid materials.

A metallic needle embedding an array of 10 temperature sensors based on fiber Bragg grating (FBG) technology (FiSens GmbH, Braunschweig, Germany) was used to measure the temperature distribution across tissue depths during heating [4,39]. The 10 FBGs with a 1 mm sensing length and edge-to-edge distance of 1 mm were inscribed in the core of a single-mode and polyimide-coated optical fiber (1550 nm wavelength operation range). The length of the array is helpful to cover the relevant portion of the tissue inside the container. The information provided by these sensors is useful to assess the required time to reach the thermal equilibrium condition of the sample with the water of the bath at each temperature step. The starting temperature for the experiment was measured to be 22 °C. An optical spectrum interrogator (Micron Optics si255, Atlanta, GA, USA, 1 pm accuracy corresponding to 0.1 °C) was used to interrogate the sensors and collect their optical output as a function of the tissue temperature.

A galvanized cylindrical container was used and filled with the tissue; further, a lid was used to prevent direct contact of water with the tissue. The lid covering the samples was manufactured to have three holes to allow the TEMPOS’s probe and FBG sensors to be inserted. The container housing the tissue was placed inside a water bath to control and tune the tissue temperature. The specifications of the water bath are as follows:

- Temperature range: 20 to 100 °C;
- Temperature fluctuation: 0.5 °C;
- Fast ramp-up: 20 to 37 °C in 10 min;
- Rated wattage: 200 W.

The accuracy of the TEMPOS is provided in the manual of the instrument: for *k*, the accuracy is 10% in the range 0.02–2.0 W/(m·K); for *D* > 0.2 mm^2^/s, the accuracy is 10%, whereas for K between 0.10 and 0.20 W/(m·K), the accuracy is 0.02 mm^2^/s; and for *C_v_* > 0.1 MJ/(m^3^·K), the accuracy is 10% [40].

**Figure 1:**
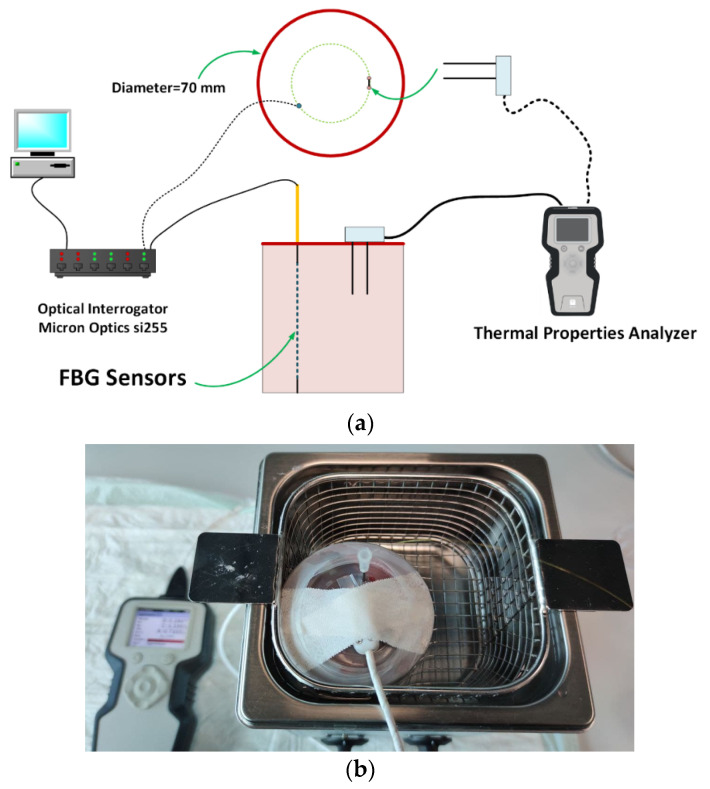
(**a**) Schematic view of the experimental setup; (**b**) picture of the container filled with liver, immersed in the temperature-controlled water bath, and including TEMPOS’s probe and needle housing FBG sensors.

Figure 1a shows the schematic view of experimental setup used to measure the tissue thermal properties; the picture of the final experimental setup is presented in Figure 1b.

Experiments were performed on porcine livers and pancreases, and calf brains provided by a local butcher. Entire portions of livers were cut in order to fill the volume of the container. Two complete porcine pancreases and half of a calf brain were used for each experiment. These specimens were wrapped in a sealed plastic bag and stored in the refrigerator at 4 °C and kept at room temperature for about two hours before the experiment. Bulk fatty tissue was removed from the pancreas before each experiment. All tissues were pruned and filled in the container to ensure a consistent tissue volume for every measurement. For each measurement, the container was connected to the TEMPOS analyzer to measure the thermal properties.

The FBGs and the TEMPOS SH-3 sensor were embedded at the same distance from the container center to ensure that they were in the same condition when the temperature increased. A needle (1.2 × 50 mm) was used to embed the FBGs into the tissue before the experiment [39].

### 2.2. Thermal Property Measurement Method

The thermal property analyzer collects data for 30 s to determine the temperature drift. When the drift is below a specific threshold (i.e., drift > 0.002 °C/s), a current is applied to the heater needle for *t_h_* (i.e., 30 s), and the temperature in the sensing needle is monitored. After 30 s, the current is shut off, and the temperature is monitored for 90 s. The monitored data are then processed by subtracting the ambient temperature and the rate of drift. In order to estimate the thermal conductivity *k* (W/(m·K)) and the thermal diffusivity *D* (mm^2^/s), Equations (2) and (3) were used to fit the measured data by means of the least squares method:

(2) $$\Delta T=\left[\frac{q}{4\pi k}\right]E_{i}\left[\frac{-r^{2}}{4Dt}\right]$$

(3) $$\Delta T=\left[\frac{q}{\pi k}\right]\left(E_{i}\left[\frac{-r^{2}}{4D\left(t-t_{h}\right)}\right]-E_{i}\left[\frac{-r^{2}}{4Dt}\right]\right)$$

In Equations (2) and (3), Δ*T* is the temperature rise at the measuring needle (°C), *q* is the heat at the heated needle (W/m), *r* is the distance from the heated needle to the measuring needle (m), *t* is time (s) and *E_i_* is the exponential integral, and it is approximated using polynomials [41]. The values of *t_h_*, *q*, *r* and *t* are available based on the probe features.

The water bath was set to a series of constant temperatures, *T_s_*, in the range from 22 to 97 °C. According to the indication of the sensors about the temperature distribution across the tissue depths, the tissue was maintained for about 1.5 h at each *T_s_* to allow the tissue to reach thermal equilibrium; after this time span, the measurement was performed. For each tissue, the temperature was increased from room temperature to about 97 °C, and the procedure was repeated on different experiments of the same fresh tissue (i.e., three livers, four brains) to include the inter-sample variability. In the case of the pancreas, the measurements were repeated on four pancreas samples until the maximum temperature of 45 °C.

#### 2.2.1. Measurement Uncertainty

For the three measured quantities, *k*, *D* and *C_v_*, and for each set temperature *T_s_*, the results are reported according to the expression in Equation (4), which follows the guidelines of the “Guide to the expression of uncertainty in measurement” [42]:

(4) $$y_{T_{s}}=\bar{y_{T_{s}}}\pm U=\bar{y_{T_{s}}}\pm k_{f}\cdot s$$

where *y* is the single thermal property, $\bar{y}$ is the arithmetic mean of the *n* measurements and *U* is the expanded measurement uncertainty; *U* is calculated by multiplying the coverage factor *k_f_* by the standard uncertainty *s*. The term *s* is expressed as the experimental standard deviation of the mean (Equation (5)):

(5) $$s=\sqrt{\frac{\sum_{i=1}^{n}{\left(y_{T_{s},i}-\bar{y_{T_{s,i}}}\right)}^{2}}{n\left(n-1\right)}}$$

The value of *k_f_* is obtained considering Student’s t-distribution, with a confidence level of 95%. The coverage factor *k_f_* is 3.18 for the brain and pancreas (*n* = 4; thus, the degrees of freedom are 3), and it is 4.30 for the liver (*n* = 3; thus, the degrees of freedom are 2).

The uncertainty in the experimental result was reported to give information about the quality of experimental data and to provide a fair comparison with other similar values or a theoretical prediction.

#### 2.2.2. Thermal Property Modeling

The thermal properties of biological tissues as a function of temperature can be described by exponential curves [18]. For this reason, the experimental data of the liver and brain were modeled by Equation (6). Here, a, b and c are the equation coefficients in the best data fitting. The least squares method was employed to obtain the coefficients of this equation by using MATLAB^®^ (MathWorks, Natick, MA, USA) [19]. The coefficient of determination (R^2^) was also evaluated to measure how well the model replicated the data.

(6) y(T)=a+b·exp(cT)

In the considered temperature range [28], thermal properties of the pancreas linearly change with temperature. Therefore, a linear equation (Equation (7)) was used to model the behavior of this tissue.

(7) (T)=aT+b

The model performance was evaluated by using the mean percentage error (MPE). The MPE was calculated using Equation (8), where $y_{exp}$ denotes the average value of the experimental data, $y_{pred}$ is the predicted data and *n* is the number of experiments in the whole temperature range:

(8) $$MPE=\frac{100}{n}\overset{n}{\underset{k=0}{\sum }}\left|\frac{y_{exp}-y_{pred}}{y_{exp}}\right|$$

## 3. Results

The thermal properties of the three different tissues with their associated uncertainties are reported. Furthermore, for each tissue, a correlation equation and its performances are presented to predict the tissue behavior.

### 3.1. Temperature Distribution in Tissue 

**Figure 2:**
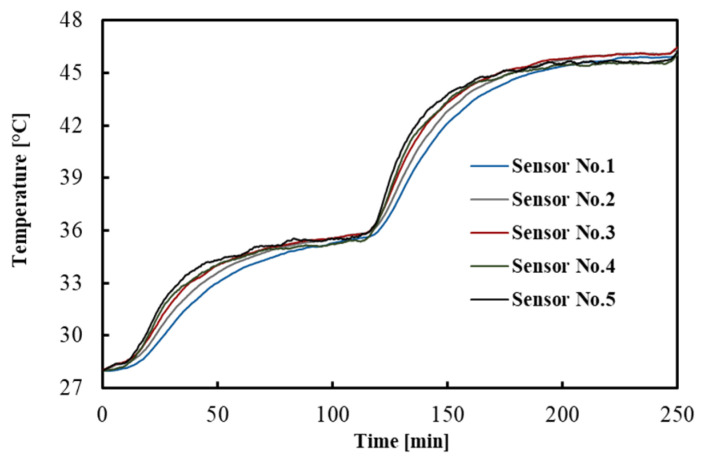
Temperature distribution across the tissue depths. This measurement refers to one of the experiments performed in the brain.

The temperatures measured by the FBGs across the tissue depths as a function of time are shown in Figure 2. For the sake of clarity, a subset of sensor responses is shown, i.e., 5 out of 10. The result indicates that after 1.5 h, the temperatures reached a constant value, which is a good criterion for the equilibrium condition.

### 3.2. Liver

**Figure 3:**
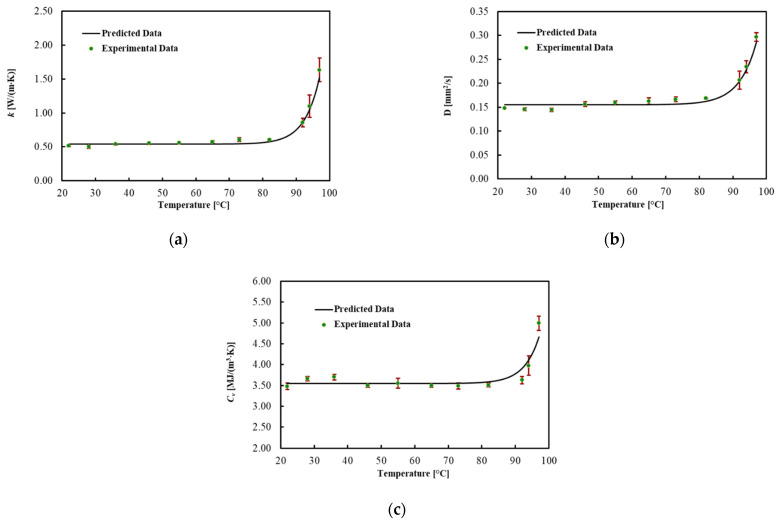
(**a**) Thermal conductivity, (**b**) thermal diffusivity and (**c**) volumetric heat capacity for ex vivo porcine livers as a function of temperature and their associated uncertainty.

The thermal properties for ex vivo porcine livers were obtained in the temperature range of about 22 up to about 97 °C for three experiment trials. The average values for each thermal property and associated uncertainty with a level of confidence of 95% are shown in Figure 3 and Table 1, for different *T_s_*. In addition, the best fitted model is presented in this figure. Thermal properties were almost constant until about 70 °C and then gradually changed until 92 °C. As the temperature rose above 92 °C, the increase in the properties with temperature became substantial. The result indicates the most considerable change for *k* and *D*, which, respectively, rose by 60% and 40% at 92 °C from the minimum value at 22 °C. *C_v_* was almost constant until 92 °C and increased by 40% up to 97 °C. As shown in Table 1 and Figure 3a–c, the uncertainty increased after 92 °C.

To describe the temperature dependency of the thermal properties, the best curves were mathematically fitted using Equation (6). The regression coefficients, the MPE and the R^2^ for each thermal property are reported in Table 2. The model fitted the data with R^2^ = 0.990 for *k*, with R^2^ = 0.978 for *D* and with R^2^ = 0.875 for *C_v_*. In addition, the MPE between the best curve and the mean values of the measured data was 5% for *k*, 4.1% for *D* and 3.2% for *C_v_*. 

### 3.3. Brain 

**Figure 4:**
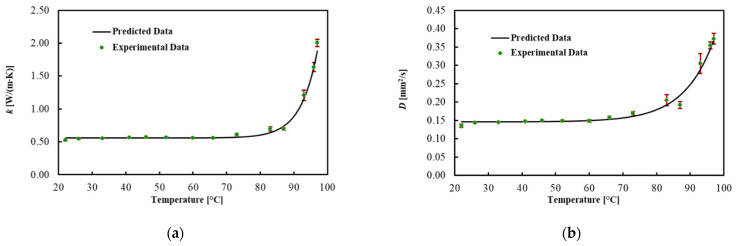

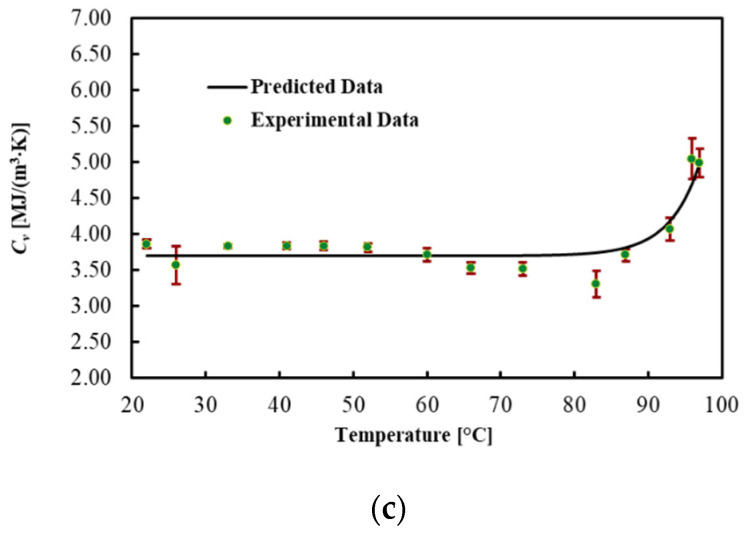
(**a**) Thermal conductivity, (**b**) thermal diffusivity and (**c**) volumetric heat capacity for ex vivo calf brains during heating.

Table 3 and Figure 4 present the average values for each thermal property, the best fitted curve and the associated uncertainty at different *T_s_*. Results show that the thermal conductivity increased by 100% in the brain up to 92 °C, while thermal diffusivity increased by 90%. However, the heat capacity did not significantly change in this temperature range. As shown in Figure 4, the significant changes in the thermal properties of the brain occurred above 92 °C. Specifically, a 4-fold increase in thermal conductivity and a 2.5-fold increase in thermal diffusivity were observed in the temperature range from 92 to 97 °C. The regression coefficients, the MPE and the R^2^ are reported in Table 4. The model fitted the data with R^2^ = 0.991 for *k*, with R^2^ = 0.984 for *D* and with R^2^ = 0.868 for *C_v_*. The MPE for *k* is 3.7%, 3.5% for *D* and 4.1% for *C_v_*.

### 3.4. Pancreas

**Figure 5:**
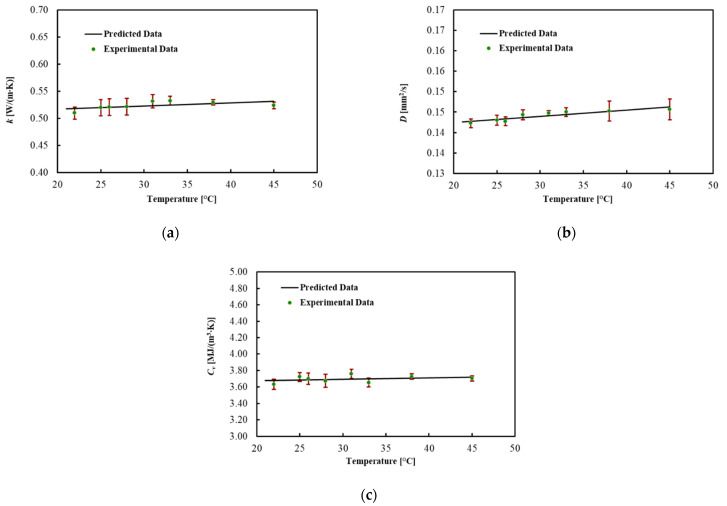
(**a**) Thermal conductivity, (**b**) thermal diffusivity and (**c**) volumetric heat capacity for ex vivo porcine pancreases during heating.

The thermal properties of the ex vivo porcine pancreatic tissue were measured by increasing the temperature from 22 °C. By increasing the temperature, a phase change, from a solid to a semi-liquid phase, was observed at around 45 °C for all four samples. For this reason, the results are shown in Table 5 and Figure 5 until 45 °C. In this range, the thermal properties of the pancreas are almost constant. The coefficients for the linear regression are presented in Table 6. The MPE for all thermal properties was lower than 1%.

## 4. Discussion

Due to the advances in diagnostic imaging and the minimally invasive nature of ablative techniques, the application of thermal procedures has raised the attention of the medical community for the treatment of tumors which typically foreshadow a poor prognosis, such as liver, brain and pancreatic cancers [43,44]. This has led to the implementation of ex vivo and preclinical studies [34,45,46] for the feasibility assessment and optimization of the procedural settings for effective tumor eradication and clinical trials evaluating the final therapeutic outcome [47,48,49,50,51,52].

Thermal properties, such as thermal conductivity, thermal diffusivity and volumetric heat capacity, are essential for determining how heat propagates in biological tissues during thermal ablation [53]. The findings of this study can be used to model the temperature-dependent changes in tissue and thus are useful for the pre-planning of thermal therapies. Indeed, changes in the thermal properties impact the heat distribution in biological tissue. The proposed equations, which are based on the measured data, could be used to determine how the temperature affects the thermal properties of various tissues. Furthermore, we used the same experimental setup and methods to measure the thermal properties of three different tissues in our research. This aspect is beneficial since it allows for a consistent comparison of different organs under similar experimental conditions. The results show the variability of the thermal properties among the three tissues when the parameters are compared at close temperatures. 

The results of this research are compared to other studies that have characterized liver tissue in Table 7. The presented comparison indicates that liver tissue results agree with the literature [18,19,20,30] and approves the used setup for an accurate measurement of liver thermal properties. In agreement with previous studies, the results report a major change for thermal conductivity and thermal diffusivity, which, respectively, increased by 60% and 40% at 92 °C from their minimum at 22 °C. The volumetric heat capacity was almost constant until 92 °C and increased by 40% up to 97 °C. This rise can be attributed to the onset of water vaporization in the tissue, with a local increase in the gas pressure and diffusion of water vapor into lower pressure areas where the temperature is lower, causing vapor condensation. In agreement with other studies, the uncertainty associated with the measurement increased at higher temperatures [20].

The brain thermal properties were presented based on a comprehensive experiment oriented to their application in the field of hyperthermia-based brain tumor treatment. To the best of our knowledge, this is the first experimental study reporting the temperature-dependent changes in the thermal properties of the brain in the mentioned temperature range. The results can be viewed as a first step toward the development of a model that can predict the outcomes of different ablation procedures. At temperatures relevant for the clinical application, such as 37 °C and 60 °C, these properties do not vary significantly compared to the liver. Indeed, at 33 °C, *k* = 0.536 ± 0.065 W/(m·K), *D* = 0.147 ± 0.017 mm^2^/s and *C_v_* = 3.83 ± 0.44 MJ/(m^3^·K); above 60 °C, k = 0.560 ± 0.064 W/(m·K), *D* = 0.158 ± 0.019 mm^2^/s and *C_v_* = 3.53 ± 0.43 MJ/(m^3^·K). Thermal necrosis occurs in tissue at temperatures higher than 60 °C, due to irreversible protein denaturation [54]; hence, the investigation of tissue properties above 60 °C is of paramount importance. At temperatures exceeding 92 °C, the thermal properties of brain tissue change dramatically. Up to 92 °C, the brain thermal conductivity increased by 100%, while thermal diffusivity increased by 90%. In this temperature range, however, the volumetric heat capacity did not vary appreciably. At temperatures exceeding 92 °C, major changes in the brain occurred. After this temperature, thermal conductivity and thermal diffusivity increased by about 4 times and 2.5 times, respectively, which can be attributed to the water vaporization in the tissue. At 97 °C, the values for the calf brain were *k* = 2.005 ± 0.253 W/(m·K), *D* = 0.373 ± 0.051 mm^2^/s and *C_v_* = 4.98 ± 0.68 MJ/(m^3^·K).

The thermal properties of the pancreas were reported up to 45 °C because at this temperature, the tissue deforms and becomes semi-liquid. This phenomenon can be ascribed to the fatty texture of this organ, considering that the melting temperature of porcine leaf fat is between 43 and 48 °C [55,56,57]. The thermal properties of the pancreas were found to be reasonably close to those found in [28]. However, the values obtained in our research are slightly higher. This difference can be ascribed to different aspects such as the tissue source and preparation, along with the different experimental approaches. At 38 °C, the thermal properties of the porcine pancreas were *k* = 0.529 ± 0.060 W/(m·K), *D* = 0.146 ± 0.006 mm^2^/s and *C_v_* = 3.70 ± 0.42 MJ/(m^3^·K).

Even though this work investigates and reports the temperature-dependent thermal properties of several tissues of interest for thermal procedures, this study presents some limitations. This work considers ex vivo animal organs, and measurements made under ex vivo conditions may differ from those of living tissue. In particular, since the liver and the brain are highly vascularized, the blood flow, volume and pressure may impact tissue thermal behavior [28,58]. Blood perfusion plays a major role in heat dissipation in living tissues, as shown by A. Bhattacharya et al., who further demonstrated the higher thermal conductivity of in vivo pig livers, mostly caused by blood perfusion [59]. However, the measurement of these properties in living models can be particularly invasive, especially in organs of difficult access, such as the pancreas and the brain. Hence, the effect of blood perfusion in the whole heat transfer phenomenon can be considered by adding a term to the heat transfer equation (Equation (1)) as a heat sink during thermal ablation [60].

Regarding the temperature values, the range of 42–100 °C is considered to be of interest for the implementation of the different techniques for cancer treatment, from hyperthermia to thermal ablation [5]. The effect of temperatures above 100 °C, which may occur in some procedures, and which are not experimentally considered in our work, has been demonstrated to cause a decrease in thermal properties [19] due to water vaporization. However, the phase change occurrence due to water vaporization may be included in the heat transfer equation (Equation (1)); in this way, the effect of the phase transition in the tissue undergoing thermal ablation could be considered [53,61].

## 5. Conclusions

The motivation of this research was to provide new data to the scientific community to be utilized in numerical modeling of thermal therapies. The thermal properties of the liver, brain and pancreas were measured as a function of temperature in steady-state heat transfer conditions. No significant thermal property changes were observed in the range 22–70 °C. In the range 70–92 °C, overall changes in the thermal properties of 50% and 90% were observed in the liver and brain, respectively. At higher temperatures (above 92 °C), approaching the water vaporization process, a sudden increase in the thermal property values was recorded. The thermal properties of the pancreas were presented at 22–45 °C, and no significant change was observed in this interval. Finally, the correlation describing the temperature dependence of the properties was proposed for each tissue to represent the trend of ex vivo tissues’ thermal properties at room temperature up to 97 °C.

## Figures and Tables

**Table 1:** Measured thermal properties for ex vivo porcine livers at different temperatures and their associated measurement uncertainty with a 95% confidence level.

Set Temperature *T_s_* (°C)	Thermal Conductivity *k* (W/(m·K))		Thermal Diffusivity *D* (mm^2^/s)		Volumetric Heat Capacity *Cv* (MJ/(m^3^·K))	
Mean	Mean	Uncertainty	Mean	Uncertainty	Mean	Uncertainty
22	0.515	0.014	0.148	0.001	3.48	0.08
28	0.504	0.027	0.146	0.004	3.66	0.05
36	0.537	0.009	0.144	0.004	3.70	0.07
46	0.550	0.014	0.156	0.005	3.50	0.04
55	0.559	0.009	0.160	0.004	3.55	0.12
65	0.571	0.017	0.163	0.007	3.50	0.05
73	0.607	0.028	0.166	0.005	3.48	0.07
82	0.603	0.015	0.168	0.002	3.51	0.05
92	0.858	0.061	0.206	0.019	3.63	0.09
94	1.099	0.167	0.235	0.013	3.98	0.24
97	1.635	0.175	0.297	0.009	4.99	0.17

**Table 2:** Regression coefficients, R^2^ and mean percentage error (MPE) of the model for liver tissue.

Thermal Property	a	b	c	MPE (%)	R^2^
Thermal Conductivity *k* (W/(m·K))	0.543	4.41 × 10^−10^	0.222	5.0	0.990
Thermal Diffusivity *D* (mm^2^/s)	0.155	4.95 × 10^−10^	0.201	4.1	0.978
Volumetric Heat Capacity *C_v_* (MJ/(m^3^·K))	3.542	1.79 × 10^−10^	0.233	3.2	0.875

**Table 3:** Measured thermal properties for ex vivo calf brains at different temperatures and their associated measurement uncertainty with 95% confidence level.

Set Temperature *T_s_* (°C)	Thermal Conductivity *k* (W/(m·K))		Thermal Diffusivity *D* (mm^2^/s)		Volumetric Heat Capacity *C_v_* (MJ/(m^3^·K))	
Mean	Mean	Uncertainty	Mean	Uncertainty	Mean	Uncertainty
22	0.524	0.010	0.136	0.005	3.86	0.06
26	0.544	0.001	0.143	0.001	3.56	0.26
33	0.553	0.004	0.145	0.001	3.83	0.03
41	0.563	0.005	0.147	0.001	3.83	0.04
46	0.574	0.006	0.149	0.001	3.83	0.06
52	0.567	0.011	0.149	0.003	3.81	0.06
60	0.560	0.007	0.149	0.003	3.71	0.09
66	0.560	0.006	0.158	0.003	3.53	0.08
73	0.611	0.016	0.170	0.005	3.52	0.09
83	0.697	0.034	0.205	0.015	3.30	0.19
87	0.696	0.017	0.192	0.009	3.71	0.09
93	1.209	0.080	0.305	0.027	4.06	0.16
96	1.635	0.069	0.354	0.009	5.04	0.28
97	2.005	0.057	0.373	0.014	4.98	0.20

**Table 4:** Regression coefficients, R^2^ and mean percentage error (MPE) of the model for brain tissue.

Thermal Property	a	b	c	MPE (%)	R^2^
Thermal Conductivity *k* (W/(m·K))	0.558	2.261 × 10^−9^	0.208	3.7	0.991
Thermal Diffusivity *D* (mm^2^/s)	0.147	9.406 × 10^−7^	0.127	3.5	0.984
Volumetric Heat Capacity *C_v_* (MJ/(m^3^·K))	3.732	9.530 × 10^−11^	0.240	4.1	0.868

**Table 5:** Measured thermal properties of ex vivo porcine pancreases at different temperatures and their associated measurement uncertainty with 95% confidence level.

Set Temperature *T_s_* (°C)	Thermal Conductivity *k* (W/(m·K))		Thermal Diffusivity *D* (mm^2^/s)		Volumetric Heat Capacity *C_v_* (MJ/(m^3^·K))	
Mean	Mean	Uncertainty	Mean	Uncertainty	Mean	Uncertainty
22	0.510	0.011	0.142	0.001	3.63	0.06
25	0.520	0.015	0.143	0.001	3.72	0.05
26	0.520	0.016	0.143	0.001	3.70	0.07
28	0.521	0.015	0.144	0.001	3.68	0.08
31	0.531	0.012	0.145	0.001	3.76	0.05
33	0.532	0.008	0.145	0.001	3.66	0.05
38	0.529	0.005	0.145	0.002	3.73	0.03
45	0.524	0.006	0.146	0.003	3.70	0.03

**Table 6:** Regression coefficients and mean percentage error (MPE) of the model for pancreas tissue.

Thermal Property	a	b	MPE (%)
Thermal Conductivity *k* (W/(m·K))	5.7 × 10^−4^	0.506	0.8
Thermal Diffusivity *D* (mm^2^/s)	1.5 × 10^−4^	0.139	0.4
Volumetric Heat Capacity *C_v_* (MJ/(m^3^·K))	1.6 × 10^−4^	3.645	0.9

**Table 7:** Comparison between obtained results for thermal properties in this work and studies that have characterized liver tissue.

Temperature (°C)	Result of this Work (Ex Vivo Porcine Liver)	Nuno P. Silva et al. [20] (Ex Vivo Ovine Liver)	Lopresto et al. [19] (Ex Vivo Bovine Liver)	Guntur et al. [18] (Ex Vivo Porcine Liver)	Choi et al. [30] (Ex Vivo Human and Porcine Liver)
Conductivity (W/(m·K))					
22	0.51	0.5	0.5	0.5	0.57
80	0.6	0.56	---	0.67	0.56
92	0.85	0.58	0.76	---	---
95	1.09	---	1.19	---	---
97	1.63	1.08	---	---	---
99	---	---	2.25	---	---
Diffusivity (mm^2^/s)					
22	3.48	3.39	3.49	3.68	3.50
80	3.51	3.37	---	---	---
90	---	3.41	3.36	4.30	3.60
92	3.63	3.55	3.84	---	---
95	3.98	---	4.17	---	---
97	4.99	5.05	---	---	---
99	---	---	7.31	---	---
Volumetric Heat Capacity (MJ/(m^3^·K))					
22	0.148	0.15	0.14	0.15	---
80	0.168	0.16	---	---	---
90	---	0.18	0.17	0.19	---
92	0.206	0.16	0.20	---	---
95	0.235	---	0.29	---	---
97	0.297	0.23	---	---	---
99	---	---	0.31	---	---

## Data Availability

Not applicable.
